# Prevalence of degenerative vertebral disc changes in elite female Crossfit athletes – a cross-sectional study

**DOI:** 10.1186/s12891-023-07071-9

**Published:** 2023-12-11

**Authors:** Mathis Wegner, Jan-Christoph Backhauß, Yannik Michalsky, Henrik Seesko, Johannes Hensler, Tim Klueter, Olav Jansen, Andreas Seekamp, Sebastian Lippross

**Affiliations:** 1 Department of Orthopedics and Trauma Surgery, University Hospital Schleswig-Holstein, Arnold-Heller-Strasse 3, Kiel, 24105 Germany; 2 Department of Radiology and Neuroradiology, University Hospital Schleswig-Holstein, Arnold-Heller-Strasse 3, Kiel, 24105 Germany

**Keywords:** Degenerative disc disease, 3.0 Tesla MRI, Crossfit, Pfirrmann grading, Female athletes

## Abstract

**Background:**

Crossfit athletes consistently recruit or transfer high levels of repetitive forces through the spine, and MRI has documented a higher rate of intervertebral disc degeneration in athletes compared with matched controls. The aim of this study was to evaluate early degenerative spinal disc changes in elite female CrossFit athletes quantified by 3.0 Tesla magnetic resonance imaging (MRI) matched with female none-athletes.

**Methods:**

In a cross-sectional single-center study 19 asymptomatic adult participants, nine German female elite Crossfit athletes and ten female participants underwent spinal MRI (3.0T). Demographic data, spinal clinical examination results and sport-specific performance parameters were collected prior to the MRI. The primary outcome was the prevalence of degenerative spinal disc changes. The secondary outcome was the grade of degeneration using Pfirrmann grading.

**Results:**

A total of 437 discs underwent spinal MRI (3.0T). The prevalence of early degenerative disc disease was not increased. Pfirrmann degenerative grade did not show significant differences among groups.

**Conclusion:**

Asymptomatic female elite Crossfit athletes do not show an increased prevalence of degenerative disc disease. Compared to a sex-matched control group, high training volume in Crossfit does not correlate to a higher incidence of degenerative disc changes in young females.

## What this study adds to existing knowledge

The findings of our study have the potential to significantly contribute to the field of sports medicine by shedding light on the relationship between Crossfit training and degenerative spinal pathologies in elite female athletes. The study shows that elite female Crossfit athletes do not experience increased spinal degeneration from high volume Crossfit training.

## Introduction

Crossfit is a strength and conditioning program that combines various forms of high-intensity functional movements such as weightlifting, gymnastics, and endurance training to improve overall fitness. The workouts are performed in a group setting and are often intense and varied, with the goal of improving overall fitness and performance [1]. Associations between competitive sports and degenerative spinal pathologies have been demonstrated in several studies [2–10]. During the 2016 Olympic Games, 10% of Olympic weightlifters were diagnosed with moderate and severe degenerative disc changes by MRI [11]. Disc degeneration is often asymptomatic and the first sign of degenerative spinal disease, followed by disc stenosis, osteophyte formation, and resulting spinal stenosis, and may be accompanied by pain and other neurological symptoms [8]. Because Olympic weightlifting and general strength training are essential components of Crossfit training, athletes apply a high level of repetitive forces to the spine, the back muscles, discs, and vertebrae. Formal exposure to extreme stress leads to the spinal injuries seen in Crossfit athletes [5]. The stress is primarily concentrated in the lower back, with the lumbar spine being the most common site of injury [12].

In contrast low back pain (LBP) is a common condition and symptom characterized by stiffness, pain, and discomfort in the lumbar region in society. 80% of adults report at least one episode of low back pain in their lifetime [13]. Acute and chronic symptoms include inflammatory conditions such as spinal stenosis or ankylosing spondylitis; mechanical problems such as injuries to the muscles, ligaments, or discs of the lower back; and degenerative conditions such as osteoarthritis or herniated discs. In terms of diagnosis, most guidelines recommend against imaging in patients with nonspecific LBP [14] because degenerative findings on MRI have no clinically significant association with LBP [15]. Therapeutic interventions include avoiding risk factors such as smoking and immobility and increasing activity and fitness levels, particularly spinal muscle strengthening [16].

This creates an ambivalence between the fact that ambitious strength training can cause degenerative spinal pathologies and the fact that it is part of the therapeutic program of LBP.

There is no clear consensus on whether Crossfit or weightlifting increases the risk of degenerative spine disease. Some studies suggest that heavy lifting and repetitive motions may increase the risk of back pain and spinal problems. Radiological changes have been identified in 84% of weightlifters with only few of them showing clinical correlation [9]. Others suggest that working and training with high weights, when performed properly, may help improve spinal health and reduce the risk of degenerative disease [17, 18]. Vadala et al. [9] showed that degenerative disc changes in a group of 13 young male weightlifters, assessed by the Pfirrmann score, were already present, although the participants were asymptomatic.

Nowadays, more and more women are practicing Crossfit. To date, there have been no studies that address spine-specific degenerative problems in female high-performance Crossfit athletes. Since Crossfit, weightlifting, and powerlifting were mostly considered male-dominated, there is a growing importance of work that more closely examines gender-specific characteristics of various strength and conditioning disciplines.

## Materials and methods

In a cross-sectional, single-center study between January and March 2023 national elite female Crossfit athletes were examined using subjective evaluation, clinical examination and MRI to provide information on degenerative disc pathology and degenerative spinal pathology.

A total of 19 female participants (age 27.9 ± 2.95 years, height 1.70 ± 0.06 m, weight 63.8 ± 7.36 kg) took part. All patients were either female Crossfit elite athletes (*n* = 9, age 26 ± 2.7 years, height 1.70 ± 0.07 m, weight 66.3 ± 7.18 kg), participating in the first national league (Fitness Bundesliga) 2022/2023 [19] or a healthy non-athletic female control group (*n* = 10, age 29.5 ± 2.9 years, height 1.70 ± 0.04 m, weight 61.5 ± 6.67 kg). Inclusion criteria were an elite fitness level defined through participation in the first German Fitness League 2022/2023, a nationwide elite Crossfit competition with eight match days throughout the year.

Participants with a history of diagnosed acute or degenerative spinal disease were excluded. One participant was excluded due to an acute cervical disc herniation. Control group participants were not included if there was a history or a current status of crossfit activity.

Athletes and control group were required to report current training status and clinical status using a questionnaire. Data on current peak performances in backsquat, deadlift, snatch, clean and jerk, and push press were assessed in the athlete group (Table 1). The questionnaire also recorded clinical symptoms related to the spine, risk factors for spine diseases, and daily habits such as smoking. In addition, participants were clinically examined and asked to complete patient-related outcome measures (PROMs) on subjective back pain (Roland and Morris Disability Questionnaire, Modified Oswestry Low Back Pain Disability Index) [11, 19] (Table 2).

**Table 1:** Personal best 1 repetition maximum of female crossfit elite athlete group. Shown in mean ± standard deviation

	Backsquat (kg)	Deadlift (kg)	Snatch (kg)	Clean & Jerk (kg)	Push-Press (kg)
1RM^a^	109 ± 14.59	128.78 ± 17.02	69.22 ± 9.34	85.72 ± 10.07	68.22 ± 8.87

^a^1 Repetition Maximum

**Table 2:** Demographic data and PROMs of crossfit elite athlete group compared to control group shown in mean ± standard deviation

	Athlete group (mean ± SD)	Control group (mean ± SD)
Age (years)	26.22 ± 2.73	29.5 ± 2.87
Height (m)	1.69 ± 0.08	1.70 ± 0.04
Weight (kg)	66.3 ± 7.18	61.5 ± 6.67
BMI (kg/m²)	22.98 ± 0.82	21.24 ± 2.08
Weekly hours of sports (h)	14.11 ± 7.83	4.33 ± 3.0
MOLBPDI^a^	4.22 ± 11.23	0.4 ± 1.33
RMDQ^b^	0.44 ± 1.33	0.11 ± 0.35

^a^Modified Oswestry Low Back Pain Disability Index

^b^ Roland and Morris Disability Questionnaire

MRI imaging of the entire spine for all participants was performed using a 3 Tesla MAGNETOM Vida (Siemens Healthineers Germany, Erlangen, Germany). The protocol included sagittal T1-weighted, T2-weighted, and short tau inversion recovery sequences for the cervical, thoracic, and lumbar spine. In addition, axial T1- and T2-weighted sequences were obtained for the cervical and lumbar spine. MRI examinations were performed by trained clinicians. The MRI examinations reviewed by radiologists experienced in the evaluation of orthopedic and traumatic spinal injuries and chronic spinal disorders, particularly disc degeneration, and were supervised by a board-certified neuroradiologist. The Pfirrmann classification system was used for the entire spine to grade disc degeneration, primarily in T2-weighted sequences (Table 3). Pfirrmann grade I - was classified as normal, grade II – III as mild, grade IV as moderate and grade V as severe pathological changes [20].

**Table 3:**
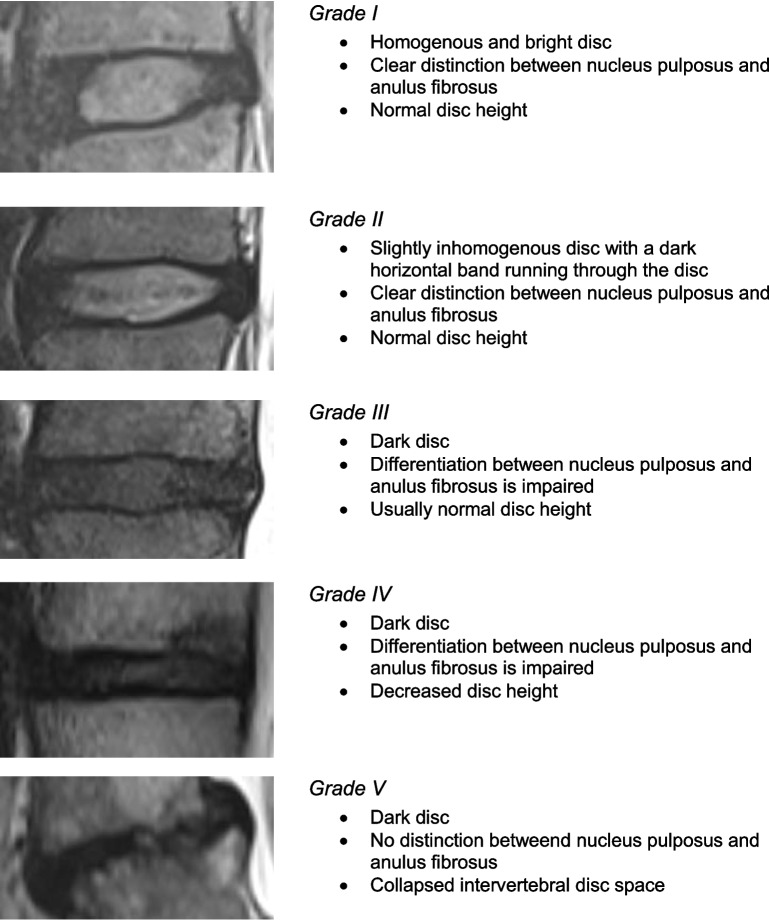
Pfirrmann grading system

Grading of disc degenerations with sagittal T2 weighted sequences

In addition, any disc herniation [21], any facet joint arthropathy [22], any high intensity zone [23], any spondylolisthesis with Meyerding > grade I [24], any Modic-type endplate change [25], any Schmorl lesion defined as focal depression [26], and any neuroforaminal narrowing [27] were recorded as findings for spinal degeneration.

### Statistical analysis

Examination results and MRI findings were presented as means with standard deviation. Significance was set at *p* < 0.05 using a Mann-Whitney test for unpaired nonparametric variables and a Fisher´s exact test for contingency tables of the additional findings. All analysis were carried out using Prism 9 for macOS Version 9.5.1.

## Results

Nineteen asymptomatic participants, nine elite German female Crossfit athletes, and ten participants matched for sex with a total of 437 discs underwent spinal MRI (3.0T). Demographic data, and sport-specific performance parameters are shown in Tables 1 and 2. The distribution of Pfirrmann classification type I-V is shown in Table 4.

**Table 4:** Absolute and relative frequency of Pfirrmann classification entered according to location and group affiliation in n (%). The athlete group contains 9 individuals, while the control group contains 10 individuals

	Cervical		Thoracic		Lumbar	
	Athlete	Control	Athlete	Control	Athlete	Control
I	3 (5.6%)	1 (1.7%)	3 (2.8%)	3 (2.5%)	0	7 (14.7%)
II	30 (55.6%)	36 (60%)	103 (95.4)	116 (96.7%)	38 (84.4%)	35 (70.0%)
III	21 (38.9%)	23 (38.3%)	1 (0.9%)	0	7 (15.6%)	4 (8.0%)
IV	0	0	1 (0.9%)	1 (0.8%)	0	4 (8.0%)
V	0	0	0	0	0	0

**Fig. 1:**
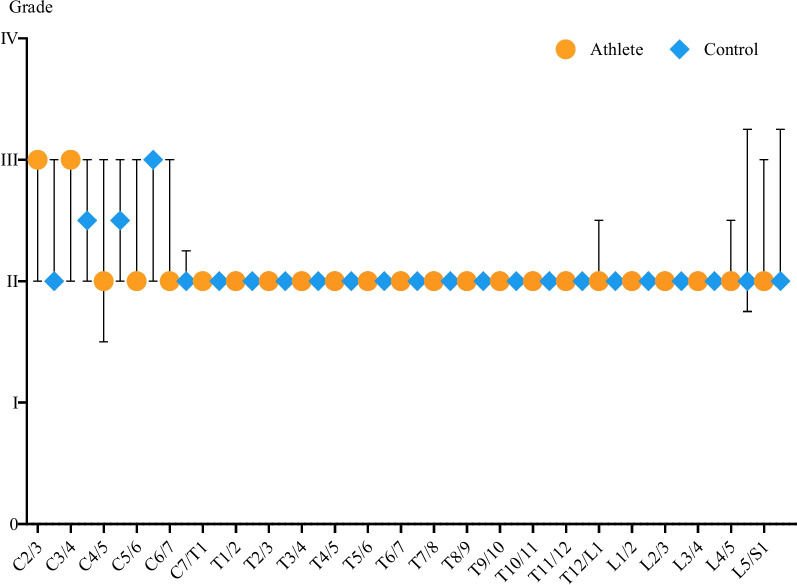
Distribution of the Pfirrmann classification system broken down to location on saggital T2-weighted images of the spine. Displayed as median with interquartil range

Distribution and severity of MRI findings are shown in Table 5. Figure 1 shows the distribution of the Pfirrmann classification broken down according to location on sagittal T2-weighted images of the spine (Fig. 1).

**Table 5:** Absolute and relative frequency of severity of MRT findings entered according to location and group affiliation in n (%). The athlete group contains 9 individuals, while the control group contains 10 individuals. Pfirrmann grade I - was classified as normal, grade II – III as mild, grade IV as moderate and grade V as severe pathological changes

	Cervical		Thoracic		Lumbar	
	Athlete	Control	Athlete	Control	Athlete	Control
Normal	3 (5,6%)	1 (1,7%)	3 (2,8%)	3 (2,5%)	0	7 (14%)
Mild	51 (94,4%)	59 (98,3%)	104 (96,3%)	116 (86,7%)	46 (100%)	39 (78,0%)
Moderate	0	0	1 (0,9%)	1 (0,8%)	0	4 (8,0%)
Severe	0	0	0	0	0	0

### Cervical spine

One hundred fourteen intervertebral discs of the cervical spine were examined. Four discs were classified as grade I (3.5%), 64 discs as grade II (56.1%), and 44 discs as grade III (38.5%) according to Pfirrmann.

In the Crossfit elite female athletes, 52 of 54 cervical spine discs showed mild degenerative changes (grade II: *n* = 30, 55.6%; grade III: *n* = 21; 38.9%), and two discs showed no signs of degenerative changes. Degenerative changes occurred mainly in the upper cervical spine (C2/C3, C3/C4) and lower cervical spine (C5/C6). The control group had mild degenerative changes in the cervical spine in 59 of 60 discs (grade II: *n* = 36, 60%; grade III: *n* = 23, 38.3%). The differences in degenerative disc changes between the athletes and the control group were not significant (Table 6).

**Table 6:** Results of Mann-Whitney testing for unpaired variables of the differences in degenerative disc changes between the athletes and the control group. Degenerative changes are presented as median Pfirrmann grade. Significance was set as *p* < 0.05

	Athlete	Control	Significance (*p*-value)
C2/3	3	2	no (0.2971)
C3/4	3	2.5	no (> 0.9999)
C4/5	2	2.5	no (0.3177)
C5/6	2	3	no (0.3698)
C6/7	2	2	no (0.3498)
C7/T1	2	2	no (0.5263)
T1/2	2	2	no (> 0.9999)
T2/3	2	2	no (> 0.9999)
T3/4	2	2	no (> 0.9999)
T4/5	2	2	no (0.4737)
T5/6	2	2	no (0.4737)
T6/7	2	2	no (> 0.9999)
T7/8	2	2	no (> 0.9999)
T8/9	2	2	no (> 0.9999)
T9/10	2	2	no (> 0.9999)
T10/11	2	2	no (> 0.9999)
T11/12	2	2	no (> 0.9999)
T12/L1	2	2	no (0.7214)
L1/2	2	2	no (> 0.9999)
L2/3	2	2	no (> 0.9999)
L3/4	2	2	no (> 0.9999)
L4/5	2	2	no (0.9639)
L5/S1	2	2	no (0.9571)
Cervical	14	14	no (0.7625)
Thoracic	24	24	no (0.7972)
Lumbar	10	10.5	no (0.9149)
Whole spine	49	49	no (0.9483)

### Thoracic spine

Two hundred twenty-eight intervertebral discs of the thoracic spine were examined. According to the Pfirrmann classification, 6 discs were classified as grade I (2.6%), 219 discs as grade II (96.1%), one disc as grade III (0.4%), and 2 discs as grade IV (0.8%).

One hundred four of 108 thoracic spine discs in female Crossfit athletes showed mild degeneration (grade II: *n* = 103; 95.4%; grade III: *n* = 1, 0.9%), three discs showed no signs of degeneration, and one disc showed moderate degenerative change. In comparison, the control group showed predominantly mild degenerative changes in 116 of 120 discs examined (grade II: *n* = 116, 96.7%; grade III: *n* = 0, 0%). One disc showed moderate degenerative change (grade IV: *n* = 1, 0.8%). Moderate degenerative changes were examined in the distal thoracic spine (T12/L1). The differences in degenerative disc changes between the athletes and the control group were not significant (Table 6).

### Lumbar spine

In the lumbar spine, 96 intervertebral discs were examined. Seven discs were classified as grade I (7.3%), 74 discs as grade II (77.1%), eleven discs as grade III (11.5%), and four as grade IV (4.2%).

Forty-six of 46 (100%) discs in elite female Crossfit athletes exhibited mild degeneration (grade II: *n* = 39, 84.8%; grade III: *n* = 7, 15.2%) in the lumbar spine. One athlete had an additional sixth lumbar vertebral body, resulting in an extra disc. In the control group, 50 discs were examined. Forty discs showed mild degeneration (grade II: *n* = 35, 70%; grade III: *n* = 4, 8%), four discs showed moderate degeneration (grade IV: *n* = 4, 8%). The most severely affected discs were located in L4/L5 and L5/S1. The differences in degenerative disc changes between the athletes and the control group were not significant (Table 6).

**Fig. 2:**
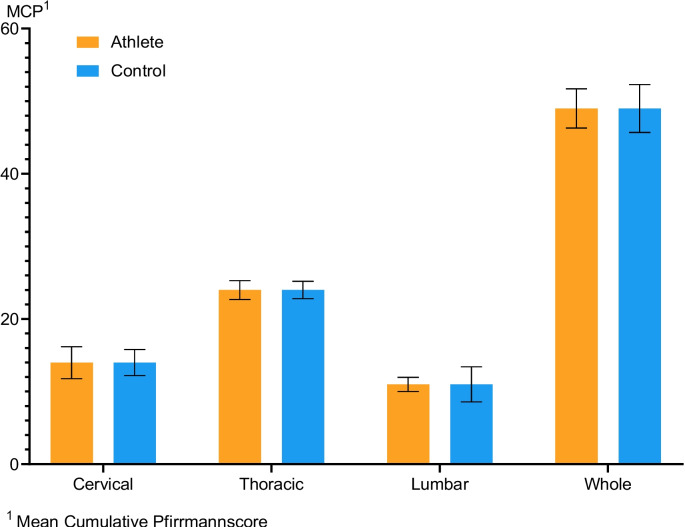
Mean cumulative Pfirrmann score (MCP) of the cervical, thoracic, lumbar and whole spine in comparison between the athlete and the control group with SD error bar

Mean cumulative Pfirrmann scores of the cervical, thoracic, lumbar and whole spine in comparison are shown in Fig. 2.

### Additional findings for spinal degeneration

In addition, incidental findings for spinal degeneration were collected in all participants. All of the German female Crossfit elite athletes studied had an asymptomatic protrusion or bulging of at least one intervertebral disc. Protrusions or bulges of intervertebral discs were found in 16 discs, predominantly in the cervical and lumbar spine (cervical spine: *n* = 6, 37.5%; lumbar spine: *n* = 6, 37.5%). In comparison, 70% of the control group had bulging or protrusion of the intervertebral discs. Pathology was observed in *n* = 10 discs (cervical spine: *n* = 2, 20%; lumbar spine: *n* = 8, 80%). The differences were not statistically significant (*p* = 0.2105). In the lumbar spine, high intensity zones were observed in two athletes and five participants. Osteochondrosis intervertebralis was detected in one disc of one athlete (Modic type I) and in two discs of one participant in the control group (Modic type I). Spinal stenosis was found in 3 Crossfit athletes, 2 in the cervical spine and one in the lumbar spine. Due to the short pedicles aetiology of spinal stenosis was diagnosed as congenital. No spinal stenosis was present in the control group. The difference was not statistically significant (*p* = 0.0867). Facet joint arthropathy in the lumbar spine was found in 7 athletes and 7 participants in the control group. The differences were not statistically significant (*p* > 0.9999). No spondylolisthesis was present in our cohort. Vertebral endplate changes defined as Schmorl nodes, were observed in 17 vertebrae in five Crossfit athletes, predominantly in the thoracic and lumbar spine. In comparison, 20 vertebrae in 4 participants in the control group had Schmorl nodes. The differences were not statistically significant (*p* = 0.6563). MRI showed scoliosis of 11° to 25° in five athletes (55.6%). There was no scoliosis in the control group. The difference was significant (*p* = 0.0108). Further findings are listed in Table 7.

**Table 7:** Comparison of additional findings for spinal degeneration of the athlete group and control group. The analysis was performed using Fisher’s exact test. Significance was set at *p* < 0.05

	Athlete (n)	Control (n)	Significance (*p*-Value)
Scoliosis	5	0	yes (0.0108)
Endplate changes	5	4	no (0.6563)
Spinal canal stenosis	3	0	no (0.0867)
Spondylarthrosis	7	7	no (> 0.9999)
Disc protrusion	9	7	no (0.2105)

**Fig. 3:**
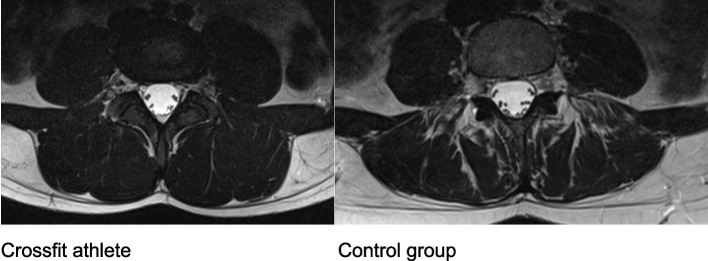
Streaky fat deposits in the autochthonous back muscles and psoas major muscle of a control participant (Goutallier 2) compared to a crossfit athlete with no visible fat infiltration and greater muscle volume (Goutallier 0)

Athletes show significantly less fat infiltration in the autochthonous back muscles and a larger muscle volume according to the Goutallier classification [28] (Fig. 3).

## Discussion

There are several data on degenerative changes of the spine in competitive athletes [2–4, 6–8, 10, 19]. In contrast, data on injury and degeneration are scarce in female strength and endurance athletes. To address this lack of research data was the rationale for our study. To our knowledge, this study is the first to examine early degenerative disc disease in female Crossfit athletes.

Regarding the cervical spine, almost all athletes in our study had some degree of disc degeneration, although they were asymptomatic. This contrasts with a study by Boden et al. who found that 25% of asymptomatic subjects under 40 years of age had some degree of cervical disc degeneration [29]. Compared with a study by Matsumoto et al., our data show significantly more degeneration in the cervical spine as they observed. In their study, degeneration was present in 17% of the discs of men and 12% of women in their twenties [30]. However, it should be noted that the two studies mentioned above did not use 3.0 Tesla MRI and therefore the quality of the data found is difficult to compare. In a retrospective MRI study conducted by Abdalkader et al. [31], it was found that 58% of the participating athletes in the 2016 Olympic Games between the ages of 20 and 30 years had degenerative findings in the cervical spine. Moderate cervical disc degeneration was most common in track and field athletes, followed by boxing. Most degenerative discs were observed in C5/C6 and C6/C7, which is consistent with our data. The higher incidence of degenerative changes in this region is probably due to the higher mobility of these spinal segments [31].

Radiological abnormalities of the thoracic and lumbar spine, a higher incidence of disc degeneration, and a direct association between disc degeneration and back pain have been found in various athletes [6, 9, 32]. Our cohort had a high incidence of mild degeneration of thoracic intervertebral discs. During the 2016 Olympic Games, 10% of weightlifters had mild, moderate, and severe degenerative changes in the lumbar spine [11]. In comparison, early degenerative lumbar disc disease in our cohort was mild. Moderate or severe degeneration was not seen in any Crossfit elite athletes.

Currently, there is no clear consensus on whether Crossfit or weightlifting increases the risk of spinal degeneration. In contrast, acute spinal pathologies, particularly in the lumbar spine, are common during crossfit training, with radicular symptoms being the most common symptom [5]. In a study by Videmann et al. in 2003, it was found that increased axial loading resulted in a significantly worse mean signal intensity score and a significantly higher mean disc bulge score than an age- and sex-matched control group [33]. In addition, Baranto et al. [3] conducted a biomechanical investigation demonstrating that the spine is vulnerable to injuries when subjected to flexion or extension loading. At this point, it should be reiterated that the present study involved asymptomatic women without currently diagnosed spinal pathology. Protrusions or bulges of the intervertebral discs were present as incidental findings in our studied group, but showed no significant differences compared with a sex-matched control group, suggesting that the radiologically observed degeneration is already present in young women, regardless of the volume or intensity of physical loading. In the context of high-volume Crossfit training, this suggests that transferring heavy loads at high frequency does not increase the incidence of spinal degenerative changes in young women.

The results of our study show that degenerative disc changes on MRI are not necessarily associated with low back pain. Our results show degenerative findings in MRI in both examined groups but a clinical association with lower back pain is missing as both groups were asymptomatic. This is consistent with a study by Kasch et al. [15], who investigated associations between common degenerative changes of the lumbar spine observed on MRI and current or future low back pain.

Looking at the underlying pathomechanisms, mechanical stress cannot be considered as the only triggering factor for degeneration. Disc degeneration is a multifactorial process, mechanical factors, metabolism and nutrition, age and genetic factors are also involved in the development of the pathology [34, 35].

The main limitations of this study are the small number of participating female athletes due to the narrow inclusion criteria, the complexity of the assessment, and the difficulty in recruiting elite female Crossfit athletes. The limited number of patients in both cohorts may not capture the full extent of the potential clinical and radiological effects of crossfit training on the spine. As a result, the study may not capture the magnitude of secondary effects and interactions. With this in mind, the data should be considered preliminary and further studies should be conducted.

Further observations can be made when analyzing the MRI images of female Crossfit elite athletes compared to the control group. Considering that participants in our cohort would generally not receive an MRI in the absence of symptoms, anatomical differences can be observed after repeated high-intensity training (Fig. 3).

Future MRI studies of the spine, including both symptomatic and asymptomatic athletes, need to be conducted to improve understanding of disc disease pathology, associations with clinical degeneration symptoms, and effects of Crossfit training on the spine. This could lead to therapeutic strategies for patients with symptomatic degenerative spine disease.

## Conclusions

To date, there have been no studies that have addressed spine-specific problems in female Crossfit elite athletes. Our data show that the prevalence of early degenerative disc disease was not increased in asymptomatic female elite Crossfit athletes. No statistically significant differences were found when compared to a sex-matched control group, suggesting that high training volume in crossfit does not correlate to a higher incidence of degenerative disc changes in young female crossfit athletes.

## Data Availability

The datasets used and analysed during the current study are available from the corresponding author on reasonable request.
